# Out of sight: chronic kidney diseases among diabetic patients attending care and follow up. Findings from pastoralist health facilities of Southern Ethiopia

**DOI:** 10.3389/fpubh.2024.1326011

**Published:** 2024-02-19

**Authors:** Eskinder Israel, Ushula Deboch Borko, Kuma Mota, Mihret Tesfaw, Tihun Feleke, Awoke Abraham, Bereket Akako, Beniyam Samuel, Amdehiwot Aynalem

**Affiliations:** ^1^School of Public Health, College of Health Science and Medicine, Wolaita Sodo University, Wolaita Sodo, Ethiopia; ^2^School of Medicine, College of Health Science and Medicine, Wolaita Sodo University, Wolaita Sodo, Ethiopia; ^3^Department of Public Health, Marie-stopes International (MSI) Ethiopia Reproductive Choices, Hawassa, Ethiopia; ^4^Department of Nursing, Hawassa College of Health Science, Hawassa, Ethiopia; ^5^Division of Maternal and Child Health, Wolaita Zone Health Department, Wolaita Sodo, Ethiopia; ^6^School of Medicine, College of Medicine and Health Science, Hawassa University, Hawassa, Ethiopia; ^7^Department of Midwifery, College of Health Science and Medicine, Dilla University, Dilla, Ethiopia; ^8^School of Nursing, College of Medicine and Health Science, Hawassa University, Hawassa, Ethiopia

**Keywords:** chronic kidney disease, diabetic patient, prevalence, pastoralists, Southern Ethiopia

## Abstract

**Background:**

In Ethiopia, the prevalence of chronic kidney disease (CKD) among the adult population ranges to 19.1%. The disease's impact has increased in low-resource settings due to a lack of knowledge about the condition and its risk factors. Diabetes is one of the numerous causes of CKD. Despite this, little was known in Ethiopia, particularly in the study area. This study aimed to identify the determinants of CKD among diabetic patients enrolled in care and follow up at pastoralist health facility of Jinka General Hospital (JGH), Southern Ethiopia, 2023.

**Methods:**

An institutional-based cross-sectional study design was conducted among 626 diabetic patients recruited through a systematic random sampling. Data was collected using a structured interviewer-administered questionnaire and entered into Epi data version 7.2 and then exported to the Statistical Package for Social Sciences (SPSS) version 25 for further analysis. Bivariate and multivariate logistic regression analyses were conducted to find eligible variables for the later analysis. Variables with *p* < 0.25 at bivariate logistic regression were selected for multivariate logistic regression analysis. The variables with *p* < 0.05 at the multivariate analysis were taken as statically significant in the final model.

**Results:**

The prevalence of CKD was 2.7% (95% CI: 1.12–6.01%). Place of residence (AOR: 4.84; 95% CI: 1.51–15.40), presence of hypertension (AOR: 5.69; 95% CI: 1.58–20.51) and family history of CKD (AOR: 6.20; 95% CI: 1.40–15.49) were factors associated with CKD among diabetes patients.

**Conclusion:**

The prevalence found in this study was low when compared with the local studies. Provision of health education to diabetic patients on preventative measures such as physical exercise is cost-effective approach. Factors associated with CKD among diabetics can be significantly mitigated by strengthening the existing NCDs prevention packages in the study area particularly and in Ethiopia generally.

## Background

Chronic kidney disease (CKD) is the severest form of kidney disease characterized by poor glomerular filtration rate (GFR) below 60 ml/min per 1.73 m^2^ or the presence of albuminuria over 3 months or abnormalities in the kidney structure (1). It is regarded as a silent killer that takes various forms from simple to advanced stage and severe health problems. Diabetes mellitus (DM) is a chronic metabolic disorder caused by either failure of pancreatic beta cells to produce an insulin or tissue resistance to use insulin or both (2). It remained as the most common cause of CKD and end-stage renal disease (ESRD) globally.

Worldwide, the prevalence of CKD is significantly rising above the optimum level and demands global urgent attention (3). Each year, more than 700 million cases of CKD and over a million people die because of CKD due to lack of access to renal replacement therapy treatment such as kidney transplant or dialysis in 2017 (4, 5). Sub-Saharan Africa (SSA), which remains the home for various non-communicable diseases (NCDs) reported hypertension and DM were primary causes of CKD (3). Despite some improvements in the prevention and treatment of NCDs, the prevalence of CKD still ranges between 2% and 41% in the SSA region (6). Various studies from African countries such as Ethiopia, South Africa, and Morocco were conducted on the magnitude of CKD and yielded different rates such as 18.2, 24.6, and 34.7% respectively (7–9). In Ethiopia, NCDs accounted for nearly 39% of annual deaths in the country each year and out of those, 10–40% of deaths were associated with diabetes and as high as 19% of the adult population had CKD in 2018 (10, 11). Poor awareness about the disease and its risk factors has been a problem for its increased prevalence in resource-limited settings including Ethiopia.

World Health Organization (WHO) report showed that 40% of total death in adults was attributed to NCDs in 2018 (12). If CKD is left untreated, it will lead to kidney failure or ESRD, increase premature death from cardiovascular disease (CVD), and reduce life expectancy. Previous studies further pointed that being low socioeconomic class, the presence of acute renal infection, health illiteracy, and unaffordable medical cost for both screening and treatment hinders the clients from seeking health care and has been the main risk factor for the high prevalence (5). In addition to this, changes in lifestyle, urbanization, and rapid growth of the population make CKD an important public health problem nowadays (7, 11). Therefore, early detection, as well as appropriate treatment of chronic diseases such as diabetes, and hypertension, can better improve renal outcomes and further slow or prevent the progression to an advanced stage (13).

Pastoralists are people who practice extensive livestock production systems as a livelihood. In Ethiopia, almost all (more than 97%) of them are clustered in the sparsely populated lowlands of the south, east and northeast making up 61% of the country's total land area. These segments of the population live in resource-limited settings (RLS) characterized by frequent droughts, famine and related manmade and natural disasters. On top of that, changes in climate conditions, frequent consumption of fats and salt and their sedentary way of life affect their health status.

Most studies conducted in different parts of the countries focused solely on urban settings, thereby overlooking pastoralist communities, confined only to CKD, and did not include DM. Despite this, limited shreds of information exist and whether the association exists among this community is not studied to date, especially among pastoralists of Southern Ethiopia. This reason pointed out to assess the problem in the light of context-specific insight to better design appropriate strategies and alleviate the health problem. Therefore, this study is primarily aimed at assessing the determinants of CKD among diabetic patients who were attending care and follow up at pastoralist health facilities of Jinka General Hospital, Southern Ethiopia.

## Methods

An institutional-based cross-sectional study design was conducted among diabetic patients who were attending care and follow-up at pastoralist health facility of southern Ethiopia namely, Jinka General Hospital (JGH) from June 01–August 30, 2023. Jinka town is the capital of South Omo zone, found on the border line to the way of Kenya and 750 km from the center of Addis Ababa. The zone is typically characterized by having socially marginalized population with the lowest and varying levels of socioeconomic status as well as dominated by so-called nomadic and pastoralist ways of life. Data from zonal administration indicated that it has a diverse ethnic group of about 21 different tribes. Jinka town has the largest governmental hospital namely Jinka General Hospital (JGH) that serves more than 100,000 populations.

All diabetic patients aged 18 years and above attending care and follow-up at JGH were the source population. All consequentively sampled diabetic patients attending care and follow-up at JGH during data collection period were study population. All diabetic patients aged 18 years and above attending care and follow-up during the data collection period and volunteered to participate were included in the study whereas, diabetic patients who were pregnant (to rule out gestational diabetes), and critically sick during data collection time were excluded from the study.

The sample size was calculated using a single population proportion formula with the following assumption;


$$\begin{array}{l} n=\frac{{\left(Z\alpha /2\right)}^{2}*p\left(1-p\right)}{d^{2}}\; \end{array}$$

Where, *n* = sample size, *Z* = 95% level of the confidence interval, *p* = 38.6%, the proportion of CKD among diabetic patients from the study conducted at Addis Ababa (11), *d* = the assumed marginal error (5%), and


$$\begin{array}{l} n=\frac{{\left(1.96\right)}^{2}\left(0.386\right)\;\left(0.614\right)}{{\left(0.04\right)}^{2}}=\;569 \end{array}$$

Through adding a 10% non-response rate (to increase the precision of the study and make more representative), the final sample size obtained was 626. We also calculated the sample size for the determinants using Epinfo software and got a small number of sample size and as a result, the largest sample size (sample size for objective 1) was taken (*n* = 626) as the final sample for this study. There were 720 diabetic patients in the hospital who were attending care and follow up. Since the source population is small (*N* = 720), all consequentively sampled diabetic patients in the selected hospital that fulfill the inclusion criteria were included in the final analysis.

## Variables

The outcome of interest (dependent variable) in this study was chronic kidney disease [presence of CKD (yes or no)]. Independent variables included sociodemographic characteristics (age, religion, sex, ethnicity, educational status, marital status, residence, monthly income, occupation), clinical characteristics (Systolic blood pressure, diastolic blood pressure, presence of hypertension, types of diabetes, family history of kidney disease, fasting blood sugar, presence of CKD), behavioral-related characteristics (alcohol habit, smoking habit, use of non-steroidal anti-inflammatory drugs (NSAIDs), and laboratory-related findings (serum creatinine level, urea level, serum sodium level, serum potassium level).

### Operational definitions

*Alcohol consumption:* Measured by the patients who have consumed greater than two alcoholic beverages per day (2).

*Previous history of alcohol consumption:* Those patients who consume more than two alcoholic beverages but have not consumed alcohol for the last 12 months before this study happens (2, 14).

*Current smoker:* Patients who have smoked more than 100 cigarettes in their lifetime and have smoked in the last 28 days before the study (15).

*Ex-smoker:* Those who have smoked more than 100 cigarettes in their lifetime but have not smoked in the last 28 days before the study (9).

*CKD:* Defined as having either the GFR cut-off value of < 90 ml/min/1.73 m^2^ or having the markers of kidney damage or both for a duration greater than 3 months (16).

*High blood glucose level:* According to an American diabetes association's (ADA) guideline, defined as random blood glucose levels >200 mg/dl and fasting blood sugar >126 mg/dl (9).

*High serum cholesterol level:* Patients with cholesterol levels >200 mg/dl during the study period (11).

### Data collection tools and procedures

A structured and validated data collection instrument was adapted from various literature conducted on similar topics previously. The data was collected using an interviewer-administered questionnaire after 2 days of training given for supervisors and data collectors on objective, process and confidentiality of the data.

To ensure the quality of data, training was given to data collectors. Four experienced bachelor holder nurses working in nearby health facilities collected the data to minimize bias and two master's holders supervised the entire data collection process. A pretest was done on 5% (*n* = 20) of the total sample size at a site other than the study site (Arbaminch General Hospital) to ensure the tools' appropriateness, simplicity, clarity, understandability, and coherence before collecting the actual data. During each day of collection, data was checked for its consistency and completeness by the supervisor.

### Data analysis

Data were entered and cleaned using EpiData version 3.5.1 and then exported to SPSS version 25, for further analysis. The prevalence of CKD was addressed by univariate analysis (through descriptive statistics such as proportions, means, and standard deviations to describe relevant variables). The association between CKD and explanatory variables was addressed using bivariate logistic regression analysis to identify eligible variables for multivariate analysis with a significance level of *p* < 0.25. Then, significant variables in bivariate logistic regression analysis were included in multivariate logistic regression analyses to identify the determinant variables. The significance was set at a *P*-value of 0.05 and model fitness was checked by using the Hosmer and Lemeshow model fitness test. Finally, odds ratios with 95% confidence intervals (CIs) were used to measure the presence and strength of association between the independent variables and CKD among diabetic patients.

### Ethical consideration

An ethical approval was obtained from Institutional review board of Arbaminch University, College of Medicine and Health Science (Ref No: 11269/14). Then, an official letter was written to Jinka General Hospital (JGH). Written informed consent was taken from each patient after presenting the objectives of the study. Written informed consent was taken from each patient after presenting a detailed explanation on the benefits and harm of the study. Moreover, before an interviewer administers the questionnaires, participants were informed about their full rights to agree or disagree and with the way to participate in the study and they were told about the possibility of withdrawing at any time during filling out the questionnaire. Each study participant was provided with written informed consent for willingness and confidentiality purposes.

## Results

### Magnitude of CKD among diabetic patients

**Figure 1:**
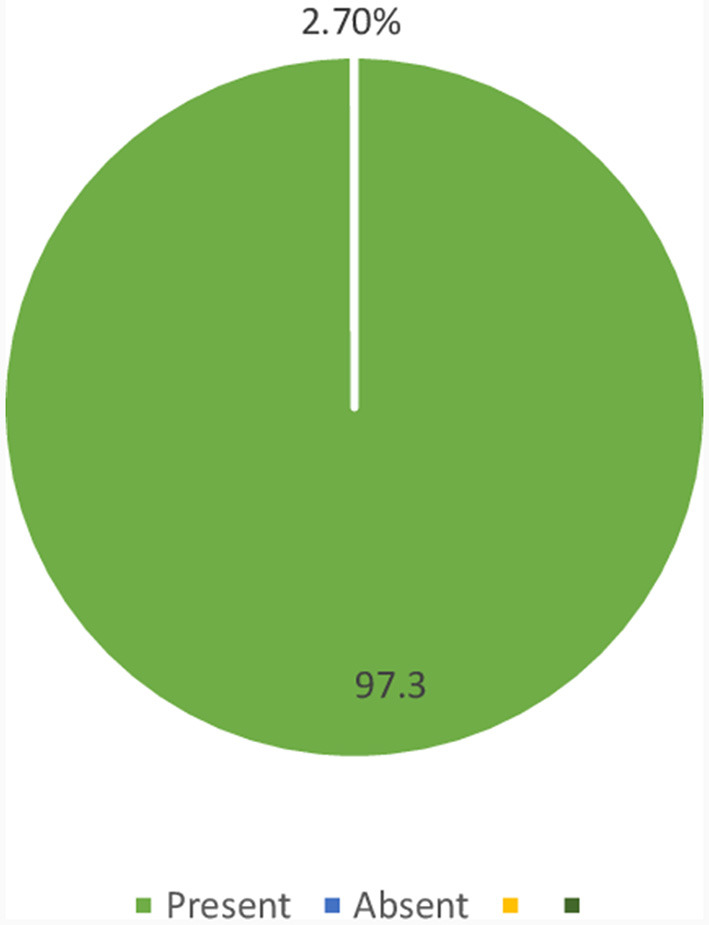
Magnitude of CKD among diabetic patients who were on follow-up at JGH, Southern Ethiopia, 2023.

In the current study, the prevalence of CKD among diabetic patients was 2.7% (95% CI: 1.12–6.01%) (Figure 1).

### Sociodemographic characteristics of the diabetic patients

A total of 626 sampled diabetic patients were participated in the final analysis making an overall response rate of 100%. Nearly half (45.4%) of the patients were aged 20–40 and more than fifty percent (52.2%) were males by sex. Regarding educational status, more than half (55.6%) of the patients were not able to read and write and the majority (85.9%) of them were married. Above half (52.2%) of the patients were from rural setting and nearly half (46.0%) had a monthly income of 1400–3000 Ethiopian Birr (ETB) (Table 1).

**Table 1:** Socio-demographic characteristics of the patients in JGH, Southern Ethiopia, 2023.

**Variables**	**Category**	**Frequency (*n* = 626)**	**Percent**
Age (year)	< 20	88	14.1
	20–40	284	45.4
	>40	254	40.6
Sex	Male	327	52.2
	Female	299	47.8
Educational level	Not able to read and write	348	55.6
	Able to read and write	131	20.9
	Primary education	94	15.0
	Secondary education	53	8.5
Marital status	Unmarried	41	6.5
	Married	538	85.9
	Divorced/separated/ widowed	47	7.5
Place of residence	Rural	327	52.2
	Urban	299	47.8
Occupation	Student	58	9.3
	Employed	214	34.2
	Unemployed	116	18.5
	Self-employed	183	29.2
	Retired	55	8.8
Monthly income (Ethiopian Birr)	< 1,500	264	42.2
	1,400–3,000	288	46.0
	>3,000	74	11.8

### Correlation coefficients—clinical characteristics of the diabetic patients

Nearly half (48.1%) of the patients had hypertension when enrolled to diabetic care. More than three-fourth (78.0%) had type II DM and above half (52.6%) had a family history of CKD. Additionally, nearly 80 percent (79.7%) had a fasting blood sugar of ≥150 mg/dl (Table 2).

**Table 2:** Correlation coefficients—clinical characteristics of the patients in JGH, Southern Ethiopia, 2023.

**Variables**	**Category**	**Frequency (*n* = 626)**	**Percent**
Presence of hypertension	Yes	301	48.1
	No	235	51.9
Types of diabetes	Type I	138	22.0
	Type II	488	78.0
Family history of chronic kidney disease	Yes	339	54.2
	No	287	45.8
Fasting blood sugar	< 150 mg/dl	127	20.3
	≥150 mg/dl	499	79.7

### Behavioral-related characteristics of the diabetic patients

More than one-third (34.7%) of the patients never used alcohol and 210 (33.5%) had never smoked in their lifetime. The majorities (88.5%) had not used any kind of non-steroidal anti-inflammatory drugs (NSAIDs) (Table 3).

**Table 3:** Behavioral-related characteristics of the patients in JGH, Southern Ethiopia, 2023.

**Variables (*n* = 400)**	**Category**	**Frequency (*n* = 626)**	**Percent**
Alcohol habit	Never used	217	34.7
	Active Users	188	30.0
	Ex-drinkers	221	35.3
Smoking habit	Never used	210	33.5
	Active users	238	38.0
	Ex-smokers	178	28.4
Use of non-steroidal Anti-inflammatory drugs	Yes	72	11.5
	No	554	88.5

### Laboratory-related characteristics of the diabetic patients

More than two-third (68.3%) of the patients had a normal serum creatinine level and above three fourth (78.9%) had a normal urea level (Table 4).

**Table 4:** Laboratory-related characteristics of the patients in JGH, Southern Ethiopia, 2023.

**Variables**	**Category**	**Frequency (*n* = 626; *n* = 400)**	**Percent**
Serum creatinine	Low	85	13.6
	Normal	396	63.3
	High	145	23.2
Urea level	Low	56	8.9
	Normal	500	78.9
	High	70	11.2

### Factors associated with chronic kidney disease among diabetic patients

Four variables were entered into bivariate and multivariate logistic regression analysis. These were a place of residence, presence of hypertension, and family history of CKD and types of DM. The multivariable logistic regression indicated that place of residence, presence of hypertension, and family history of CKD was found to be associated with chronic kidney disease among diabetic patients.

The odds of CKD among DM patients from urban areas were four times higher when compared with patients from rural areas (AOR: 4.84; 95% CI: 1.51–15.40). Likewise, hypertensive patients were over five times more likely to develop CKD than those who were not hypertensive (AOR: 5.69; 95% CI: 1.58–20.51). Patients with a family history of chronic kidney disease were six times more likely to have chronic kidney disease than their counterparts (AOR: 6.20; 95% CI: 1.40–15.49) (Table 5).

**Table 5:** Bivariate and multivariable logistic regression analysis of CKD among diabetic follow-up patients attending JGH, Southern Ethiopia, 2023.

**Variables**	**Category**	**CKD among DM patients**		**COR (95% CI)**	**AOR (95% CI)**
		**Overall (*N* = 626)**	**Positive (%)**		
Presence of hypertension	No	316	3 (0.5)	1	1
	Yes	310	14 (2.2)	4.93 (1.40–17.34)^*^	5.69 (1.58–20.51)^**^
Place of residence	Rural	325	3 (0.5)	1	1
	Urban	301	14 (2.2)	3.62 (1.16–11.23)^*^	4.84 (1.51–15.40)^**^
DM types	Type I	139	6 (1.0)	1	1
	Type II	487	11 (1.8)	0.51 (0.18–1.41)^*^	0.43 (0.16–2.01)
Family history of CKD	No	285	2 (0.3)	1	1
	Yes	341	15 (2.4)	6.51 (1.47–28.71)^*^	6.20 (1.51–15.40)^**^

^*^Variables statistically significant at *p* < 0.25, ^**^variables statistically significant at *p* < 0.05, AOR; Adjusted Odds Ration, COR; Crude Odds Ration; ^*^p < 0.05 (significant).

## Discussion

In this study, the prevalence of CKD among diabetic patients was 2.7% (95% CI: 1.12–6.01%). Place of residence, presence of hypertension, and family history of CKD were found to be associated with CKD among diabetic patients.

The rate found in this study was lower than most studies conducted in Ethiopia [Gondar (21.8%) (17), Jimma (26.1%) (14), Mekelle (22.1%) (18)], and outside [sub-Saharan African (18.2%) (19), Spain (27.9%) (20), Mediterranean area (34.1%) (21), United States of America (39.6%) (22) and Japan (42.3%) (23)]. The higher rate in countries outside Ethiopia could be due to the high prevalence of NCDs in general adult population and difference in nutritional habit (nutritional changes) among sampled population in the mentioned countries. Furthermore, the consumption of ultra-processed food and drinks (junk foods, more fats and oils) and decrease in regular physical activity leads to various cardiovascular disease and type 2 DM that further increases the chance of getting CKD. Additionally, difference in study population contributed to observed variation as this study only considered pastoralists.

The odds of CKD among DM patients from urban areas were four times higher when compared with patients from rural areas. This is in line with the scientific justification that CKD patients living in urban areas are more exposed to various non-communicable diseases due to their lifestyle changes (physical inactivity because of long time sitting for office jobs) and consumption of more saturated fats (24). In addition to this, urban population was less likely to use fruits and vegetables as compared with rural population. This finding is found to be consistent with the study conducted in the States of Palestine and Greece (25, 26).

Likewise, hypertensive patients were five times more likely to develop CKD than their counterparts. This goes with evidence in the field that hypertension can be taken as both a cause and effect of CKD and further contributes to its fast progression. It is also believed that as the eGFR level declines, the incidence as well as the severity of hypertension increase and vice versa (27, 28). High blood pressure can decrease blood supply to the vital organs including kidney which results in stopping of function to remove waste and other fluid from the body or bloodstream, which in turn further complicates the kidney. This statement is consistent with the findings from the Tigray region (18, 29). In the current study, nearly half (48.1%) of the patients with CKD have high blood pressure, which might have increased the chance that kidney disease will get worse (30). Various studies showed that hypertension remains the most important risk factor for CKD and whenever there is hypertension, glomerular filtration dramatically declines (31, 32).

In our study, patients with a positive family history of CKD were six times more likely to have chronic kidney disease than their counterparts. This is due to the evidence that if there is CKD or DM or different types of NCDs or cancer in one family, an individual with higher relativeness to that family is highly exposed as compared with non-familial relative (33, 34). Another justification for this might be CKD more affects immediate and near relatives who shared more genes through mutation that increases the risk of getting kidney problem either to immediate child or family. This statement goes with the studies conducted in Korea and Japan (35, 36).

Our study is strong as it used both primary and secondary data to collect as many information as possible to identify the determinants of CKD among diabetic patients attending care and follow up at pastoralist health facility. However, it has several limitations. Firstly, since the study used cross sectional study design, the causal association would not be inferred. Secondly, the use of secondary data resulted in incompleteness of some data. Thirdly, the study might have been affected by recall, and desirability bias due to the study design. We suggest that future studies should take these limitations into account to improve the robustness of studies on similar topics. Despite this, the study tried to see the main determinants of CKD at its best.

## Conclusion/recommendation

The prevalence found in this study was low when compared with the local studies. Provision of health education to diabetic patients on preventative measures such as physical exercise is cost-effective approach. Factors associated with CKD among diabetics can be significantly mitigated by strengthening the existing NCDs prevention packages in the study area particularly and in Ethiopia generally.

## Data availability statement

The raw data supporting the conclusions of this article will be made available by the authors, without undue reservation.

## Ethics statement

The studies involving humans were approved by Arbaminch University, College of Medicine and Health Science with Ref No: 11269/14. The studies were conducted in accordance with the local legislation and institutional requirements. The participants provided their written informed consent to participate in this study.

## Author contributions

EI: Conceptualization, Data curation, Formal analysis, Funding acquisition, Investigation, Methodology, Project administration, Resources, Software, Supervision, Validation, Visualization, Writing – original draft, Writing – review & editing. UB: Conceptualization, Formal analysis, Investigation, Methodology, Visualization, Writing – review & editing. KM: Conceptualization, Data curation, Funding acquisition, Methodology, Resources, Validation, Writing – review & editing. MT: Data curation, Funding acquisition, Software, Supervision, Validation, Visualization, Writing – review & editing. TF: Conceptualization, Funding acquisition, Methodology, Software, Validation, Writing – review & editing. AAb: Data curation, Investigation, Methodology, Validation, Visualization, Writing – review & editing. BA: Data curation, Investigation, Project administration, Supervision, Visualization, Writing – review & editing. BS: Data curation, Investigation, Project administration, Visualization, Writing – review & editing. AAy: Data curation, Funding acquisition, Investigation, Project administration, Software, Writing – review & editing.
